# Dissociation Between Subjective Sensory Reactivity and Visual Perceptual Sensitivity in Autistic and Non‐Autistic Adults: A Brief Report

**DOI:** 10.1002/brb3.70865

**Published:** 2025-09-21

**Authors:** Caroline Candy, Declan Ryan, Elizabeth Milne, Abigail Dickinson

**Affiliations:** ^1^ Semel Institute for Neuroscience University of California Los Angeles California USA; ^2^ Department of Psychology University of Michigan Ann Arbor Michigan USA; ^3^ Department of Psychology University of Sheffield Sheffield UK

**Keywords:** autism spectrum disorder, orientation discrimination, psychophysical thresholds, sensory processing, visual perception

## Abstract

**Background:**

Autistic individuals frequently report atypical sensory experiences, typically assessed using self‐ or proxy‐report questionnaires such as the Sensory Profile. However, it remains unclear whether these subjective reports reflect differences in low‐level perceptual sensitivity, with previous studies yielding mixed results.

**Objective:**

We aimed to determine whether self‐reported sensory responsiveness, assessed via the Adult Sensory Profile (ASP), is associated with low‐level visual perceptual sensitivity (orientation discrimination thresholds) in autistic and non‐autistic adults.

**Methods:**

Thirty‐two autistic and thirty‐two neurotypical adults completed a visual orientation discrimination task and the ASP. Spearman correlations and Bayesian analyses quantified associations between orientation discrimination thresholds and ASP sensory scores across and within diagnostic groups.

**Results:**

Autistic adults reported significantly greater sensory differences than neurotypical adults across all ASP quadrants. However, orientation discrimination thresholds were not significantly associated with ASP scores within either group or across the full sample. Bayesian analyses provided anecdotal to moderate evidence supporting no association between self‐reported sensory experiences and orientation discrimination performance.

**Conclusion:**

Our findings suggest that self‐reported sensory differences, as captured by the ASP, do not reflect variations in low‐level visual perceptual sensitivity. These results reinforce the importance of multilevel assessment frameworks to better understand the complex and varied sensory experiences reported by autistic individuals.

## Graphical abstract

We assessed sensory processing at different levels in autistic adults using questionnaires (high level) and a visual psychophysical task (low level). Elevated self‐reported sensory difficulties did not correspond to orientation‐discrimination thresholds, revealing a dissociation between subjective questionnaire responses and objective measures of perceptual sensitivity.

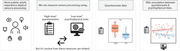

## 1 Introduction

Autism spectrum disorder (ASD) is a neurodevelopmental condition characterized by differences in social communication and restricted, repetitive behaviors, including atypical sensory responses (American Psychiatric Association 2013). Sensory differences in autism are most commonly measured using self‐ or proxy‐report questionnaires (DuBois et al. 2017), which indicate that up to 95% of autistic individuals experience atypical sensory processing (Baranek et al. 2006; Ben‐Sasson et al. 2019; Kern et al. 2007; Leekam et al. 2007; Tomchek and Dunn 2007). However, while questionnaires provide valuable insights into daily sensory experiences, they primarily capture higher level behavioral and affective responses.

Recent taxonomies emphasize that sensory responsivity assessed by self‐report questionnaires (reflecting behavioral reactions to sensory stimuli) and sensory sensitivity assessed via psychophysical tasks (reflecting low‐level perceptual processing) represent distinct, though potentially overlapping, aspects of sensory processing (He et al. 2023; Ward 2019). For example, He et al. (2023) highlight that the responsivity captured by questionnaires differs meaningfully from perceptual sensitivity, which is typically assessed using psychophysical tasks that measure an individual's ability to detect or discriminate subtle differences in sensory input. Investigating whether these low‐level perceptual sensitivities align with self‐reported sensory experiences may offer important insights into the mechanisms underpinning sensory differences in autism. If perceptual sensitivity (i.e., being more sensitive to low‐level sensory input) directly contributes to the sensory experiences reported by autistic individuals, we might expect reliable correlations between psychophysical task performance and self‐report measures such as the Adult Sensory Profile (ASP).

Despite the intuitive appeal of a link between perceptual sensitivity and subjective sensory experiences, empirical studies have yielded inconsistent findings. Some studies report positive associations, for example, between greater visual detection sensitivity (based on spatial frequency, but not contrast) and higher sensory responsivity scores (Sapey‐Triomphe et al. 2023), or between tactile perceptual sensitivity and sensory responsivity in autistic children (but not adults) (Quinde‐Zlibut et al. 2020). However, other studies have found associations in the opposite direction, with autistic individuals who endorse greater sensory difficulties on questionnaires showing evidence of reduced perceptual sensitivity on tactile (Powell et al. 2022) and auditory psychophysical tasks (Kuiper et al. 2019). Finally, several studies have found no association between perceptual sensitivity (auditory and tactile) and self‐reported sensory traits in autism (Dwyer et al. 2022), echoing findings from non‐autistic samples and supporting the idea that these two levels of processing may be at least partially dissociable (Schulz and Stevenson 2022).

These mixed findings may, in part, reflect differences in the sensory modality or psychophysical task used across studies, including visual, auditory, and tactile measures. Within the visual domain, some evidence suggests a link between perceptual sensitivity and self‐reported sensory responsivity, particularly for detection thresholds based on spatial frequency (Sapey‐Triomphe et al. 2023). To build on this work, we aimed to test whether a similar relationship exists for another basic visual feature: orientation discrimination (OD). OD refers to the ability to detect small differences in the tilt of visual stimuli and represents a fundamental aspect of early‐stage visual processing, akin to contrast or spatial frequency.

If findings of associations between self‐reported sensory responsivity and heightened perceptual sensitivity extend across visual domains, we would expect lower OD thresholds (indicating greater visual sensitivity) to be associated with higher ASP scores, the most widely used tool for assessing sensory processing in autism (DuBois et al. 2017). However, given evidence from the same autistic sample showing no association between sensory responsivity and contrast detection thresholds (Sapey‐Triomphe et al. 2023), as well as findings of no relationship between visual thresholds and self‐reported sensitivity in neurotypical (NT) adults (Schulz and Stevenson 2022), we adopted an exploratory approach supported by Bayesian statistics. This allowed us to evaluate the strength of evidence for or against an association between OD thresholds and self‐reported sensory responsivity, both across the full sample and within each diagnostic group.

## 2 Methods

### 2.1 Participants

This brief report focuses on a subset of participants from a previously published study of OD (Dickinson et al. 2016), including the 33 autistic and 34 NT adults who also completed the self‐report Sensory Profile questionnaire. Three participants (one autistic, two NT) were excluded due to extreme OD threshold values (> 1.5 × interquartile range), resulting in a final sample of 64 participants (32 autistic, 32 NT; see Table 1 for demographic characteristics).

**TABLE 1:** Participant demographics and sensory measures.

	**Autistic adults** **(*N* = 32)**	**NT adults** **(*N* = 32)**	**Group comparison** ***p* value**
Age (years)	34.25 (14.00) [18, 67]	26.41 (10.58) [18, 65]	0.014
Sex (% female)	25% (8/32)	34% (11/32)	0.673
Nonverbal cognition (WASI *T* score)	62.65 (5.64) [51, 72]	59.45 (8.29), [29, 69]	0.081
ADOS CSS (*n* = 24)	5.67 (2.46) [1–9]		
Orientation discrimination threshold	5.81 (2.13) [2.04, 9.58]	6.57 (1.85) [3.35, 10.23]	0.131
Sensory profile			
Sensory sensitivity	47.66 (9.61) [28, 70]	36.81 (8.76) [23, 63]	< 0.001
Low registration	41.75 (7.91) [24, 60]	32.72 (7.04) [21, 52]	< 0.0001
Sensation avoiding	49.56 (11.11) [29, 67]	35.66 (8.63) [24, 58]	< 0.0001
Sensation seeking	38.75 (7.32) [22, 54]	48.69 (7.65) [34, 68]	< 0.0001

Autistic participants were recruited from an existing participant database or through a local NHS neurodevelopmental service providing adult autism diagnoses. All participants had a confirmed clinical diagnosis established by a qualified clinician. ADOS scores were available for 24 of the 32 participants with autism (see Table 1). Full details on diagnostic ascertainment are provided in Dickinson et al. (2016). NT participants reported no personal or family history of autism in their first‐degree relatives and were recruited from a volunteer research database. All participants had normal or corrected‐to‐normal vision. Nonverbal cognitive abilities were assessed using the Matrix Reasoning subtest from the Wechsler Abbreviated Scale of Intelligence (WASI) (Weschler 1999). The study received ethical approval from the local research ethics committee, and all participants provided informed written consent in accordance with the Declaration of Helsinki.

### 2.2 Psychophysical Task

OD thresholds were measured using a two‐alternative forced‐choice adaptive staircase procedure, fully described in Dickinson et al. (2014). Participants judged whether a target grating, presented after a reference grating (oriented 45° clockwise from vertical), was rotated clockwise or counterclockwise relative to the reference. Thresholds were established using four randomly interleaved staircases (two clockwise, two counterclockwise) following a one‐up three‐down procedure (converging on ∼79% accuracy). Threshold values were averaged across the last eight reversals from each staircase (Dickinson et al. 2016)

### 2.3 Sensory Profile

Participants completed the self‐report version of the ASP (Dunn 1999), a 60‐item questionnaire assessing frequency of sensory‐related behaviors. Each item in the ASP assesses the frequency of sensory‐related behaviors on a scale from 1 to 5, where 1 = *behavior seldom occurs* and 5 = *it almost always occurs*. Responses from the ASP are categorized into four quadrants that reflect different sensory processing characteristics: low registration, sensory sensitivity, sensation seeking, and sensation avoiding. Primary analyses used scores from all four ASP quadrants and were repeated using quadrant scores derived from visual modality items only and the visual domain score. ASP data were collected on the same day as the OD task.

### 2.4 Statistical Analyses

All analyses were conducted in R (version 4.3.1). Group differences in demographic variables (age, sex, and nonverbal IQ), ASP scores, and OD thresholds were assessed using independent‐samples *t*‐tests and chi‐square tests (Table 1).

To investigate associations between sensory features and OD thresholds, we first examined Spearman correlations separately within the autism and NT groups. To test whether the strength of these associations differed by group, we compared correlation coefficients using Fisher's *r*‐to‐*z* transformation, implemented via the cocor package (Diedenhofen and Musch 2015). We computed Bayes factors (BF_01_) using the BayesFactor package (Morey and Rouder 2022) to quantify evidence in favor of the null hypothesis (i.e., no difference in associations between groups). When there was at least anecdotal evidence for the null (BF_01_ > 1), we collapsed across groups and examined associations in the full sample.

For these full‐sample analyses, we again used Spearman correlations alongside Bayes factors (BF_01_) to assess evidence for the absence of an association between ASP scores and OD thresholds. Bayes factors were interpreted according to established conventions: BF_01_ < 1 indicates no support for the null, values between 1–3 indicate anecdotal evidence, and values > 3 indicate moderate evidence for the null (Wagenmakers et al., 2018). Exploratory analyses included visual‐specific ASP scores (i.e., the visual domain and visual‐item‐based quadrant scores). All primary and exploratory analyses were repeated using partial Spearman correlations controlling for age and nonverbal IQ, implemented via the ppcor package (Kim 2015). Finally, we replicated all analyses in participant subsamples, which matched more closely on age and nonverbal IQ to rule out potential confounding effects.

## 3 Results

**FIGURE 1:**
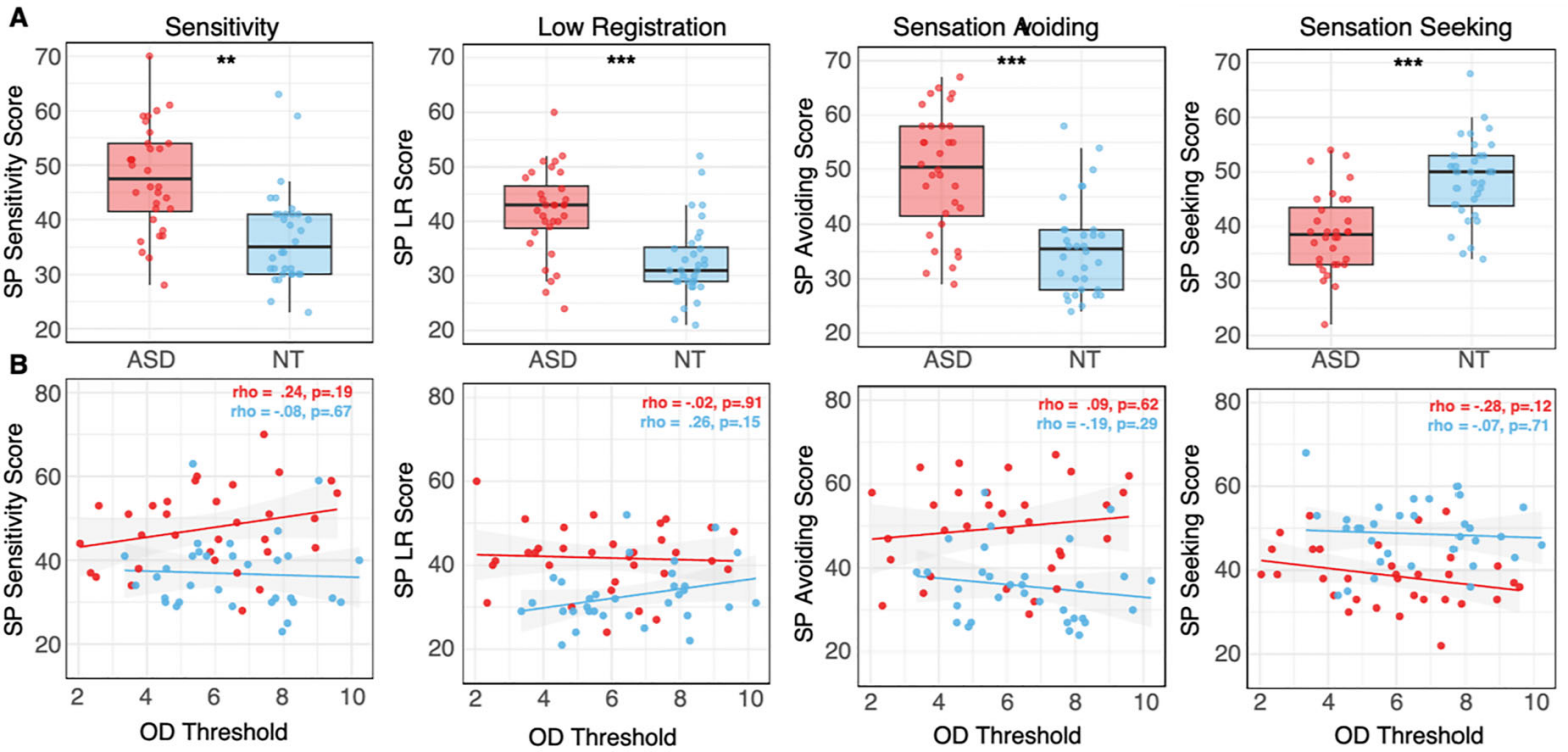
(A) Box plots show group differences in sensory profile scores across each of the four ASP quadrants (sensory sensitivity, low registration, sensation seeking, and sensation avoiding). (B) Scatter plots illustrate the relationship between oblique orientation discrimination thresholds and the four ASP quadrants. There was no significant relationship between orientation discrimination thresholds and ASP quadrant scores for autistic (red) or NT participants (blue). ***p < 0.0001, **p < 0.001.

ASP scores differed significantly between autistic and NT adults across all four quadrants (Table 1; Figure 1A). Autistic adults had significantly higher scores sensitivity (*t*(61.47) = 4.72, *p* < 0.001), low registration (*t*(61.16) = 4.82, *p* < 0.001), and sensation avoiding (*t*(58.43) = 5.59, *p* < 0.001) ASP quadrant scores, and significantly lower seeking quadrant scores (*t*(61.89) = −5.31, *p* < 0.001). OD thresholds were slightly lower in autistic (*M* = 5.81; SD = 2.13) compared to NT adults (6.57; 1.85), but did not significantly differ between groups (*t*(60.76) = −1.53, *p* = 0.131; see Table 1).

Group‐specific analyses revealed no significant associations between OD thresholds and any ASP quadrant within either group (autistic adults: *p* > 0.12; non‐autistic adults: *p* > 0.15; Table 2; Figure 1B). Fisher's *r*‐to‐*z* comparisons indicated no significant differences in the strength of these associations between groups (all *p* > 0.22). Bayesian analyses further supported the null hypothesis, with BF_01_ = 2.68–3.94, providing anecdotal to moderate evidence in favor of no group differences in the observed associations.

**TABLE 2:** Associations between orientation discrimination thresholds and sensory profile quadrants.

**ASP quadrant**	**Autism**	**NT**	**Group comparison**	**Full sample**	**Bayes factor**
	rho (*p*)	rho (*p*)	*z* (*p*), BF_01_	rho (*p*)	(BF_01_)
Sensory sensitivity	0.24 (0.19)	−0.08 (0.67)	1.22 (0.22), 2.68	−0.03 (0.82)	3.52
Low registration	−0.02 (0.91)	0.26 (0.15)	−1.10 (0.27), 3.09	0.02 (0.89)	3.50
Sensation avoiding	0.09 (0.62)	−0.19 (0.29)	1.09 (0.28), 3.12	−0.14 (0.28)	2.68
Sensation seeking	−0.28 (0.12)	−0.07 (0.71)	−0.85 (0.40), 3.94	−0.01 (0.96)	3.40

Given the absence of meaningful associations or group differences, we then collapsed across diagnostic groups and tested for associations in the full sample. OD thresholds were not significantly associated with any ASP quadrant scores in the combined sample (Table 2). Bayesian analyses further supported the absence of an association, with Bayes factors (BF_01_ = 2.68–3.52) providing anecdotal to moderate evidence in favor of the null hypothesis. Exploratory analyses of visual‐specific ASP scores similarly revealed no evidence of group differences in associations or relationships between ASP scores and OD thresholds (see Table S1). These findings remained consistent when controlling for age and nonverbal IQ (Table S2) and when analyses were repeated in subsamples more closely matched on age (Table S3) and nonverbal IQ (Table S4). Across all analyses, there was no evidence of associations between ASP scores and OD thresholds in either group or the combined sample. In each case, Bayesian results (BF_01_ > 1) consistently supported the null hypothesis, indicating no reliable relationship between ASP scores and OD thresholds.

To ensure results were not driven by differences in age or nonverbal IQ distributions, we conducted supplementary analyses using closely matched subsamples. For age, we implemented a pairwise matching procedure in which each autistic participant was matched to a NT participant within ±10 years. This yielded two age‐matched groups (*n* = 24 per group) with no significant age difference (ASD: *M* = 29.0, SD = 11.9; NT: *M* = 28.4, SD = 11.4; *t*(45) = 0.17, *p* = 0.86). We repeated this approach for nonverbal IQ using WASI *T*‐scores, matching each ASD participant to an NT participant within ±10 points. This resulted in 28 participants per group with comparable scores (ASD: *M* = 62.4, SD = 5.77; NT: *M* = 61.4, SD = 5.33; *t*(54) = 0.67, *p* = 0.50). All primary analyses were rerun within these matched subsamples, yielding consistent patterns of results (See Tables S3 and S4).

## 4 Discussion

This study examined the relationship between low‐level perceptual responsivity, as measured by OD thresholds, and self‐reported sensory experiences in autistic and NT adults. As expected, we observed clear group differences in ASP scores. Consistent with previously reported patterns and meta‐analytic findings (van den Boogert et al. 2022), autistic adults in our sample showed elevated sensory sensitivity, low registration, and sensation avoiding scores, and reduced sensation seeking scores compared to NT adults. Although OD thresholds were generally lower in autistic adults (in line with findings in the full sample [Dickinson et al. 2016]), group differences were not statistically significant in the current subsample.

Although there were clear differences between groups in self‐reported sensory responsivity, OD thresholds were not significantly linked to ASP scores within either autistic or NT adults, nor across the combined sample. Bayesian analyses further supported the absence of a relationship, providing anecdotal to moderate evidence for the null hypothesis. Moreover, no significant group differences were observed in the strength of associations, suggesting that the lack of association between OD thresholds and self‐reported sensory traits was consistent across autistic and non‐autistic adults.

These findings contribute to a growing body of evidence suggesting that subjective sensory experiences and objective measures of perceptual sensitivity reflect distinct components of sensory processing (Dwyer et al. 2022; Schulz and Stevenson 2022). Although some studies have reported associations (both positive and negative) between questionnaire‐based and psychophysical measures in autistic children and adults (Kuiper et al. 2019; Powell et al. 2022; Quinde‐Zlibut et al. 2020; Sapey‐Triomphe et al. 2023), these relationships are often inconsistent, sometimes within the same sample. For example, Sapey‐Triomphe et al. (2023) found that ASP scores were associated with visual sensitivity measured using a spatial frequency detection task, but not with a contrast detection task, highlighting the importance of the specific perceptual feature being assessed. Similarly, Quinde‐Zlibut et al. (2020) found that lower tactile detection thresholds (indicating greater sensitivity) were linked to higher sensory responsivity scores in autistic children, but not in adults. Powell et al. (2022) also reported that significant associations were limited to one of several tactile tasks used, emphasizing the task‐specific nature of these effects.

Overall, the limited and inconsistent associations suggest that sensory challenges in autism are unlikely to be explained solely by increased low‐level sensory input or basic perceptual sensitivity. Instead, other mechanisms may be involved. For example, Ide et al. (2019) found that greater sensory difficulties reported on the ASP in autistic adults were associated with enhanced ability to discriminate the temporal order of tactile stimuli, but not with lower tactile detection thresholds. This suggests that more complex perceptual judgments, particularly those involving temporal integration, may be more relevant for understanding sensory experiences in autism than basic detection thresholds alone. The lack of consistent and direct relationships between sensory sensitivity and responsivity measures also highlights the need to understand higher level cognitive, affective, and contextual factors that may shape how low‐level perceptual processing translates into real‐world behavioral responses (Ashburner et al. 2013; Sibeoni et al. 2022).

The gap between the specificity of psychophysical tasks and the broader scope of questionnaire‐based measures highlights a limitation of the present study, which relied on a single psychophysical measure (OD) to represent perceptual sensitivity. We also used a single self‐report questionnaire (the ASP), which, although widely used to assess sensory differences in autism, may lack the specificity needed to capture experiences directly linked to the perceptual mechanisms targeted by psychophysical tasks. While future research may benefit from incorporating multiple psychophysical tasks, obtaining reliable psychophysical thresholds can be time‐intensive, even for a single aspect of a sensory modality. An alternative strategy may involve developing more targeted self‐report questions that align closely with specific perceptual functions. For example, in the context of OD, individuals could be asked whether they notice when objects are misaligned or when pictures appear slightly tilted in daily life. Finally, the relatively modest sample size in this study may have limited our ability to detect subtle effects or interactions. Larger, multimodal studies will be essential for mapping the associations between different levels of sensory processing in autism.

In conclusion, we found no significant association between OD thresholds and ASP scores, consistent with a dissociation between objectively measured perceptual sensitivity and self‐reported sensory experiences. Alongside prior mixed findings, this highlights that while low‐level perceptual differences may contribute to sensory experiences in autism, they are likely intertwined with cognitive, affective, and contextual factors that vary across individuals and add to the complexity of sensory processing (Ashburner et al. 2013; Sibeoni et al. 2022). Future research should use multimodal assessment strategies that pair targeted psychophysical tasks with questionnaire items more closely aligned to the specific sensory features being measured. This may help narrow the gap between subjective and objective measures and clarify how specific perceptual differences relate to real‐world sensory experiences in autism. Such approaches are essential for uncovering the mechanisms that drive individual differences in sensory processing and for informing more tailored, effective supports and interventions that target the specific aspects of sensory processing that may be distressing or disruptive for some autistic individuals.

## Author Contributions

**Caroline Candy**: investigation, writing – original draft, writing – review and editing. **Declan Ryan**: investigation, writing – original draft, writing – review and editing, data curation. **Elizabeth Milne**: conceptualization, investigation, writing – original draft, methodology, writing – review and editing, supervision. **Abigail Dickinson**: conceptualization, investigation, writing – original draft, methodology, visualization, writing – review and editing, formal analysis, data curation, supervision, resources.

## Conflicts of Interest

The authors declare no conflicts of interest.

## Peer Review

The peer review history for this article is available at https://publons.com/publon/10.1002/brb3.70865.

## Supporting information




**Supplementary Tables**: brb370865‐sup‐0001‐Tables.docx

## Data Availability

Data sharing not applicable to this article as no datasets were generated or analyzed during the current study.
