# Supplementary material for: Dissociation Between Subjective Sensory Reactivity and Visual Perceptual Sensitivity in Autistic and Non‐Autistic Adults: A Brief Report
Source: Brain Behav. 2025 Sep 21;15(9):e70865. doi: 10.1002/brb3.70865 (PMC12451066; doi:10.1002/brb3.70865)
Supplement: Supplementary file 1 — Supplementary Tables: brb370865‐sup‐0001‐Tables.docx [file BRB3-15-e70865-s001.docx]

|  |  | **Autism** | | **Control** | |  | **Group Differences in Associations** | | |  | **Full Sample** | | |
| --- | --- | --- | --- | --- | --- | --- | --- | --- | --- | --- | --- | --- | --- |
|  |  | rho | p | rho | p |  | z | p | BF01 |  | rho | p | BF01 |
| *Visual Quadrant Scores* | |  |  |  |  |  |  |  |  |  |  |  |  |
|  | Sensory Sensitivity (Visual) | 0.23 | 0.2 | -0.01 | 0.96 |  | 0.94 | 0.35 | 3.64 |  | 0.07 | 0.6 | 2.98 |
|  | Low Registration (Visual) | -0.08 | 0.67 | 0.27 | 0.13 |  | -1.37 | 0.17 | 2.22 |  | -0.001 | 1 | 3.25 |
|  | Sensation Avoiding (Visual) | 0.02 | 0.92 | -0.24 | 0.18 |  | 1.01 | 0.31 | 3.38 |  | -0.18 | 0.15 | 1.76 |
|  | Sensation Seeking (Visual) | -0.3 | 0.1 | -0.04 | 0.84 |  | -1.04 | 0.3 | 3.3 |  | -0.008 | 0.95 | 3.36 |
| Visual Domain Score | | 0.14 | 0.45 | -0.004 | 0.98 |  | 0.55 | 0.58 | 4.87 |  | -0.01 | 0.92 | 3.14 |

Supplementary Table 1.

|  |  | **Autism** | | **Control** | |  | **Group Differences in Associations** | | |  | **Full Sample** | | |
| --- | --- | --- | --- | --- | --- | --- | --- | --- | --- | --- | --- | --- | --- |
|  |  | rho | p | rho | p |  | z | p | BF01 |  | rho | p | BF01 |
| *Sensory Profile Quadrant Scores* | |  |  |  |  |  |  |  |  |  |  |  |  |
|  | Sensory Sensitivity | 0.2 | 0.3 | -0.21 | 0.27 |  | 1.56 | 0.12 | 1.65 |  | -0.08 | 0.55 | 3.28 |
|  | Low Registration | -0.17 | 0.38 | 0.2 | 0.3 |  | -1.39 | 0.16 | 2.11 |  | -0.06 | 0.63 | 2.87 |
|  | Sensation Avoiding | 0.03 | 0.86 | -0.29 | 0.12 |  | 1.25 | 0.21 | 2.55 |  | -0.2 | 0.14 | 2.17 |
|  | Sensation Seeking | -0.36 | 0.05 | -0.07 | 0.71 |  | -1.15 | 0.25 | 2.87 |  | -0.03 | 0.8 | 2.78 |
| *Visual Quadrant Scores* | |  |  |  |  |  |  |  |  |  |  |  |  |
|  | Sensory Sensitivity (Visual) | 0.26 | 0.18 | -0.06 | 0.77 |  | 1.18 | 0.24 | 2.76 |  | 0.07 | 0.59 | 3.58 |
|  | Low Registration (Visual) | -0.17 | 0.38 | 0.16 | 0.42 |  | -1.23 | 0.22 | 2.62 |  | -0.09 | 0.48 | 2.61 |
|  | Sensation Avoiding (Visual) | -0.05 | 0.8 | -0.24 | 0.21 |  | 0.73 | 0.47 | 4.27 |  | -0.22 | 0.1 | 1.33 |
|  | Sensation Seeking (Visual) | -0.24 | 0.22 | -0.07 | 0.73 |  | -0.65 | 0.51 | 4.5 |  | -0.004 | 0.98 | 2.88 |
| Visual Domain Score | | 0.09 | 0.63 | -0.08 | 0.68 |  | 0.66 | 0.51 | 4.48 |  | -0.08 | 0.56 | 2.79 |

Supplementary Table 2. Controlling for Age and WASI T score.

|  |  | **Autism** | | **Control** | |  | **Group Differences in Associations** | | |  | **Full Sample** | | |
| --- | --- | --- | --- | --- | --- | --- | --- | --- | --- | --- | --- | --- | --- |
|  |  | rho | p | rho | p |  | z | p | BF01 |  | rho | p | BF01 |
| *Sensory Profile Quadrant Scores* | |  |  |  |  |  |  |  |  |  |  |  |  |
|  | Sensory Sensitivity | 0.25 | 0.23 | -0.08 | 0.72 |  | 1.09 | 0.28 | 2.71 |  | 0.009 | 0.95 | 3.00 |
|  | Low Registration | 0.16 | 0.46 | 0.1 | 0.66 |  | 0.22 | 0.83 | 4.79 |  | 0.04 | 0.77 | 2.87 |
|  | Sensation Avoiding | 0.09 | 0.68 | -0.16 | 0.46 |  | 0.81 | 0.42 | 3.52 |  | -0.14 | 0.33 | 2.58 |
|  | Sensation Seeking | -0.14 | 0.52 | 0.18 | 0.4 |  | -1.03 | 0.3 | 2.88 |  | 0.16 | 0.27 | 2.12 |
| *Visual Quadrant Scores* | |  |  |  |  |  |  |  |  |  |  |  |  |
|  | Sensory Sensitivity (Visual) | 0.37 | 0.07 | 0.006 | 0.98 |  | 1.26 | 0.21 | 2.23 |  | 0.16 | 0.27 | 1.25 |
|  | Low Registration (Visual) | 0.29 | 0.17 | 0.23 | 0.28 |  | 0.2 | 0.84 | 4.8 |  | 0.17 | 0.25 | 1.8 |
|  | Sensation Avoiding (Visual) | 0.04 | 0.86 | -0.16 | 0.44 |  | 0.67 | 0.5 | 3.92 |  | -0.16 | 0.28 | 2.51 |
|  | Sensation Seeking (Visual) | -0.14 | 0.51 | 0.13 | 0.54 |  | -0.89 | 0.37 | 3.3 |  | 0.13 | 0.39 | 2.49 |
| Visual Domain Score | | 0.34 | 0.1 | 0.1 | 0.65 |  | 0.83 | 0.41 | 3.47 |  | 0.12 | 0.42 | 1.98 |

Supplementary Table 3 – Restricted age sample.

|  |  | **Autism** | | **Control** | |  | **Group Differences in Associations** | | |  | **Full Sample** | | |
| --- | --- | --- | --- | --- | --- | --- | --- | --- | --- | --- | --- | --- | --- |
|  |  | rho | p | rho | p |  | z | p | BF01 |  | rho | p | BF01 |
| *Sensory Profile Quadrant Scores* | |  |  |  |  |  |  |  |  |  |  |  |  |
|  | Sensory Sensitivity | 0.13 | 0.52 | -0.09 | 0.64 |  | 0.78 | 0.44 | 3.91 |  | -0.06 | 0.66 | 3.09 |
|  | Low Registration | -0.04 | 0.86 | 0.33 | 0.09 |  | -1.33 | 0.18 | 2.2 |  | 0.03 | 0.84 | 3.25 |
|  | Sensation Avoiding | 0.05 | 0.8 | -0.23 | 0.24 |  | 1.01 | 0.31 | 3.18 |  | -0.16 | 0.23 | 2.08 |
|  | Sensation Seeking | -0.27 | 0.17 | -0.06 | 0.76 |  | -0.76 | 0.45 | 3.96 |  | 0.02 | 0.9 | 3.31 |
| *Visual Quadrant Scores* | |  |  |  |  |  |  |  |  |  |  |  |  |
|  | Sensory Sensitivity (Visual) | 0.24 | 0.22 | 0.04 | 0.82 |  | 0.7 | 0.48 | 4.13 |  | 0.09 | 0.51 | 2.65 |
|  | Low Registration (Visual) | 0.06 | 0.76 | 0.27 | 0.16 |  | -0.78 | 0.44 | 3.92 |  | 0.05 | 0.72 | 3.07 |
|  | Sensation Avoiding (Visual) | 0.04 | 0.85 | -0.26 | 0.18 |  | 1.07 | 0.28 | 2.98 |  | -0.19 | 0.15 | 1.84 |
|  | Sensation Seeking (Visual) | -0.26 | 0.19 | 0.03 | 0.88 |  | -1.03 | 0.3 | 3.11 |  | 0.04 | 0.76 | 3.28 |
| Visual Domain Score | | 0.23 | 0.24 | 0.05 | 0.79 |  | 0.65 | 0.52 | 4.29 |  | 0.03 | 0.86 | 3.31 |

Supplementary Table 4. Restricted WASI sample.
